# Evaluation of the efficacy of labor induction with vaginal misoprostol in a low-risk pregnant women population

**DOI:** 10.1590/1806-9282.20240132

**Published:** 2024-07-19

**Authors:** Letícia Sampaio Vilas-Boas, Marcos Paulo Ribeiro Sanches, Edward Araujo, Alberto Borges Peixoto, Rosiane Mattar, Leandra Rejane Rodrigues dos Santos, David Baptista da Silva Pares, Sue Yasaki Sun

**Affiliations:** 1 Universidade Federal de São Paulo, Escola Paulista de Medicina, Department of Obstetrics – São Paulo (SP), Brazil.; 2 Amparo Maternal Hospital, Service of Obstetrics – São Paulo (SP), Brazil.; 3 Universidade de Uberaba, Mario Palmério University Hospital, Service of Gynecology and Obstetrics – Uberaba (MG), Brazil.; 4 Universidade Federal do Triângulo Mineiro, Department of Obstetrics and Gynecology – Uberaba (MG), Brazil.

**Keywords:** Labor induction, Misoprostol, Maternity hospital, Bishop

## Abstract

**OBJECTIVE:**

The aim of this study was to evaluate the success rate and predictors of labor induction using vaginal misoprostol in a low-risk pregnant women population.

**METHODS:**

A prospective cohort study was carried out with 196 pregnant women. Groups 2 and 4 of the Robson Classification admitted for induction of labor with vaginal misoprostol (25 μg tablets every 6 h, up to 4 tablets, for a maximum of 24 h). The success of labor induction was considered the achievement of vaginal delivery. Binary logistic regression was used to determine the best predictors of successful induction of labor with vaginal misoprostol.

**RESULTS:**

Of all the pregnant women analyzed, 140 (71.4%) were successful and 56 (28.6%) were unsuccessful. Pregnant women who achieved successful induction had a higher number of pregnancies (1.69 vs. 1.36, p=0.023), a higher number of deliveries (0.57 vs. 0.19, p<0.001), a higher Bishop score (2.0 vs. 1.38, p=0.002), and lower misoprostol 25 μg tablets (2.18 vs. 2.57, p=0.031). No previous deliveries [x^2^(1)=3.14, odds ratio (OR): 0.24, 95% confidence interval (CI): 0.10–0.57, R^2^ Nagelkerke: 0.91, p=0.001] and the presence of one previous delivery [x^2^(1)=6.0, OR: 3.40, 95% CI: 1.13–10.16, R^2^ Nagelkerke: 0.043, p=0.029] were significant predictors of successful induction of labor with vaginal misoprostol.

**CONCLUSION:**

A high rate of labor induction success using vaginal misoprostol in a low-risk population was observed, mainly in multiparous and with gestational age>41 weeks. No previous delivery decreased the success of labor induction, while one previous delivery increased the success of labor induction.

## INTRODUCTION

Labor induction is defined as the artificial induction of labor in a pregnant woman whose gestational age is within the limits of fetal viability and who has no signs of active labor^1^. Its main indication is to ensure maternal and fetal well-being^2^, and it is mainly used in pregnancies of more than 41 weeks gestation^3^.

Rates of induced labor have been increasing over the years, with North American literature reporting an increase of more than 100% since 1990^4^. In developed countries, the proportion reaches about 25% of all births. In low- and middle-income countries, induction rates are typically lower, but in some places they can still approach those of high-income countries^5^.

Success in inducing labor depends on several factors and is more likely in multiparous and younger women, and those with a lower body mass index (BMI)^6^. Some biochemical markers can also predict this success, such as fetal fibronectin and IGFBP-1^1^. However, the condition of the cervix before the onset of labor remains the most important predictor^6^.

There are pharmacological and mechanical alternatives to cervical ripening. Pharmacological options include prostaglandin analogs such as E1 (misoprostol) or E2 (dinoprostone). Misoprostol has the advantage of being a cheap, accessible drug that can be stored at room temperature. However, prostaglandin analogs are contraindicated in patients with uterine scarring due to the associated risk of tachysystole and uterine rupture. In these cases, cervical ripening can be performed with a transcervical balloon, in which a Foley tube is inserted through the internal opening of the cervix and the balloon is inflated with 30–50 mL of volume^7^.

The International Federation of Gynaecology and Obstetrics (FIGO) recommendation for induction of labor with misoprostol is 25 μg vaginally every 6 h or orally every 2 h^8^. However, there are few studies that directly evaluate the recommended dose and duration of use of this drug. Repeated administration may prolong the latent phase of labor, which is associated with higher rates of cesarean section, chorioamnionitis, endometritis, and uterine atony^9^.

The purpose of this study was to evaluate the success rate and predictors of labor induction using vaginal misoprostol in a low-risk pregnant women population.

## METHODS

A prospective cohort study was conducted in the Amparo Maternal Hospital, a low-risk maternity hospital in the city of São Paulo, Brazil, between February and April 2022. This study was approved by the Ethics Committee of the Federal University of São Paulo (CAAE: 54185521000005505).

The inclusion criteria were pregnant women in Groups 2 and 4 of the Robson Classification admitted for induction of labor with vaginal misoprostol. The exclusion criteria were fetal malformations, uterine myomatosis, fetal death, and women not fluent in Portuguese. The participants were divided into two groups: (1) success—pregnant women who had a vaginal delivery and (2) unsuccess—pregnant women who had a caesarean section. Robson Classification Group 2 includes nulliparous, singleton pregnancy ≥37 weeks, and induced or cesarean section before labor, and Group 4 includes multiparous (excluding previous cesarean section), singleton pregnancy ≥37 weeks, and induced or cesarean section before labor^10^.

The care of the pregnant women followed the Amparo Maternal protocol for induction of labor, based on the current recommendations of the FIGO and the protocol of the Municipality of São Paulo. It consists of the vaginal introduction of misoprostol 25 μg tablets every 6 h, up to 4 tablets, for a maximum duration of 24 h.

During the study period, a book was provided to record pregnant women admitted for induction of labor and the respective Bishop score rates in each of the two inpatient rooms at the hospital. This record allowed us to identify the patients who were eligible to participate in the study. The researchers visited the maternity ward every day and, after the patients agreed and signed the informed consent form, they collected data on their care during labor induction and delivery.

The data were analyzed using SPSS version 20.0 (SPSS Inc., Chicago, IL, USA) and Prisma GraphPad 7.0 (GraphPad Software, San Diego, CA, USA). Quantitative variables were subjected to the D'Agostino and Pearson normality test. Parametrically distributed variables were presented as mean and standard deviation. Non-parametrically distributed variables were presented as median, 25th percentile, and 75th percentile. Categorical variables were described as absolute and percentage frequencies and presented in tables and graphs. Differences between categorical variables and their proportions were analyzed using the chi-square test. The effect of the groups on the continuous variables was analyzed using the Student's t-test (parametric distribution) or the Mann-Whitney test (non-parametric distribution). Binary logistic regression was used to determine the best predictors of successful induction of labor with vaginal misoprostol. The significance level adopted for all tests was p<0.05.

## RESULTS

In our study, we evaluated 196 cases of pregnant women who underwent labor induction with vaginal misoprostol. Of all the pregnant women analyzed, 140 were successful and 56 were unsuccessful. Among the patients who successfully induced labor, 44 also used oxytocin in the induction/conduction of labor, while in the group of unsuccessful patients, 20 used oxytocin. The clinical maternal/neonatal characteristics and the process of induction of labor of the entire population included in the study are identified in Table 1.

**Table 1:** Clinical maternal/neonatal characteristics of the study population.

Variable		Included cases (196)
Maternal age (years)		24.0 (21.0–29.0)
Body mass index (kg/m^2^)		29.0 (26.0–31.8)
Number of pregnancies		1.59 (0.93)
Number of deliveries		0.46 (0.80)
Nulliparous		69.9% (137/196)
At least one previous delivery		30.1% (59/196)
Gestational age (weeks)		40.3 (39.0–41.0)
Comorbidities		
	Diabetes mellitus	9.7% (19/196)
	Arterial hypertension	7.1% (14/196)
	Oligohydramnios	0.5% (1/196)
	Hypothyroidism	2.6% (5/196)
	Urinary tract infection	0.5% (1/196)
	Other	5.1% (10/196)
	None	74.5% (146/196)
Type of delivery		
	Vaginal	68.3% (134/196)
	Forceps	2.6% (5/196)
	Cesarean section	29.1% (57/196)
	APGAR score at 5th min	9.0 (9.0–10.0)
Birth weight (g)		
	<2,500	5.1% (10/195)
	2,500–2,999	23.6% (46/195)
	3,000–3,499	46.7% (91/195)
	3,500–4,000	21.0% (41/195)
	>4,000	3.1% (6/195)
	Postpartum hemorrhage	1.03% (2/194)
	Neonatal intensive care unit admission	8.2% (16/194)
Indication of labor induction		
	Diabetes mellitus	5.6% (11/196)
	Arterial hypertension	6.7% (13/196)
	Premature rupture of ovular membranes	34.2% (67/196)
	Gestational age ≥41 weeks	46.4% (91/196)
	Other	7.1% (14/196)
	Bishop score at admission	1.82 (1.4)
	Total number of misoprostol 25 μg tablets	2.3 (1.15)
	Oxytocin use	35.0% (64/183)
	Success of labor induction	71.4% (140/196)
	Time until delivery (h)	20.0 (12.0–28.0)

Mean (standard deviation); median (25th–75th percentile); percentage (absolute number/total number of cases analyzed).

Pregnant women who achieved successful induction had a significantly higher mean number of pregnancies (1.69 vs. 1.36 pregnancies, p=0.023) and deliveries (0.57 vs. 0.19 deliveries, p<0.001) than pregnant women who were unsuccessful in inducing labor with vaginal misoprostol. There was a significant association between induction success and type of delivery (p<0.001). Pregnant women with successful induction of labor had a higher mean Bishop score (2.0 vs. 1.38, p=0.002) and lower misoprostol 25 μg tablets used (2.18 vs. 2.57, p=0.031) than those with unsuccessful induction of labor. The time between the use of the first misoprostol tablet and delivery was shorter in pregnant women with successful induction compared to those with unsuccessful induction of labor (18.0 vs. 25.0 h, p<0.001) (Table 2).

**Table 2:** Clinical characteristics of pregnant women who induced labor with vaginal misoprostol.

		Success of labor induction (140)	Unsuccess of labor induction (56)	p
Maternal age (years)		24.0 (21.0–28.3)	25.0 (21.0–29.3)	0.523†
Body mass index (kg/m^2^)		29.0 (26.6–31.6)	29.0 (27.0–32.3)	0.551†
Number of pregnancies		1.69 (0.99)	1.36 (0.72)	0.023∂
Number of deliveries		0.57 (0.85)	0.19 (0.58)	<0.001∂
Nulliparous		62.9% (88/140)	87.5% (49/56)	<0.001†
At least one previous delivery		37.1% (52/140)	12.5% (7/56)	<0.001
Gestational age (weeks)		40.1 (39.0–41.0)	40.8 (39.7–41.2)	0.034†
Comorbidities				
	Diabetes mellitus	9.3% (13/140)	10.7% (6/56)	0.747ƒ
	Arterial hypertension	6.4% (9/140)	8.9% (5/56)	
	Oligohydramnios	0.7% (1/140)	0.0% (0/56)	
	Hypothyroidism	3.6% (5/140)	0.0% (0/56)	
	Urinary tract infection	0.7% (1/140)	0.0% (0/56)	
	Other	4.3% (6/140)	7.1% (4/56)	
	None	75.0% (105/140)	73.2% (41/56)	
Type of delivery				
	Vaginal	95.7% (134/140)	0.0% (0/56)	<0.001ƒ
	Forceps	3.6% (5/140)	0.0% (0/56)	
	Cesarean section	0.7% (1/140)	100% (56/56)	
	APGAR score at 5th min	9.0 (9.0–10)	9.0 (9.0–10)	0.944∂
	APGAR score at 5th min <7	0.71% (1/140)	0.0% (0/55)	>0.999
Birth weight (g)				
	<2,500	6.4% (9/140)	1.8% (1/55)	0.313ƒ
	2,500–2,999	20.7% (29/140)	30.9% (17/55)	
	3,000–3,499	49.3% (69/140)	40.0% (22/55)	
	3,500–4,000	20.7% (29/140)	21.8% (12/55)	
	>4,000	2.1% (3/140)	5.5% (3/55)	
	Postpartum hemorrhage	1.4% (2/139)	0.0% (0/55)	0.371ƒ
	Neonatal intensive care unit admission	7.9% (11/140)	9.3% (5/54)	0.594ƒ
Indication of labor induction				
	Diabetes mellitus	5.7% (8/140)	5.4% (3/56)	0.327ƒ
	Arterial hypertension	5.7% (8/140)	8.9% (5/56)	
	Premature rupture of ovular membranes	37.1% (52/140)	26.8% (15/56)	
	Gestational age ≥41 weeks	42.9% (60/140)	55.4% (31/56)	
	Other	8.6% (12/140)	3.6% (2/56)	
	Bishop score at admission	2.0 (1.39)	1.38 (1.34)	0.002∂
	Total number of misoprostol 25 μg tablets	2.18 (1.10)	2.57 (1.23)	0.031∂
	Oxytocin use	31.9% (44/138)	44.4% (20/45)	0.303ƒ
	Time until delivery (h)	18.0 (12.0–26.0)	25.0 (16.0–33.5)	<0.001†

∂ Student's t mean (standard deviation)

† Mann-Whitney median (25th percentile–75th percentile)

ƒ Chi-square percentage (absolute number/total number of cases analyzed). p<0.05.

A binary logistic regression model was created to assess whether the number of previous deliveries, BMI ≥30 kg/m^2^, Bishop's score ≤5, and Bishop's score ≥6 were predictors of successful induction of labor with vaginal misoprostol. It was found that no previous deliveries [x^2^(1)=3.14, odds ratio (OR): 0.24, 95% confidence interval (CI): 0.10–0.57, R^2^ Nagelkerke: 0.91, p=0.001] and the presence of one previous delivery [x^2^(1)=6.0, OR: 3.40, 95%CI: 1.13–10.16, R^2^ Nagelkerke: 0.043, p=0.029] were significant predictors of successful induction of labor with vaginal misoprostol. No previous delivery decreased the odds of successful induction of labor by 0.24 times, while one previous delivery increased the odds of successful induction of labor by 3.40 times. The presence of two previous deliveries (p=0.058), three previous deliveries (p=0.670), BMI ≥30 kg/m^2^ (p=0.797), Bishop's score ≤5 (p=0.515), and Bishop's score ≥6 (p=0.515) were not significant predictors of successful induction of labor with vaginal misoprostol (Table 3).

**Table 3:** Odds ratio for successful induction of labor using vaginal misoprostol considering the number of deliveries, body mass index, and Bishop score.

	OR	95% CI	p
No previous delivery	0.24	0.10–0.57	0.001
One previous delivery	3.40	1.13–10.16	0.029
Two previous deliveries	4.20	0.95–18.83	0.058
Three or more previous deliveries	1.61	0.17–14.80	0.670
BMI ≥30 kg/m^2^	0.88	0.35–2.19	0.797
Bishop score ≤5	2.52	0.15–41.1	0.515
Bishop score ≥6	0.40	0.02–6.43	0.515

CI: confidence interval; OR: odds ratio; BMI: body mass index; Binary logistic regression. p<0.05.

A weak significant negative correlation (r=0.29, p<0.0001) was observed between the Bishop score and the number of vaginal misoprostol tablets used. A weak but significant negative correlation (r=0.28, p<0.0001) was also observed between the Bishop score and time to delivery.

## DISCUSSION

Vaginal misoprostol was the most effective option for cervical preparation compared with oxytocin, dinoprostone (prostaglandin E2), and placebo, without increasing cesarean section rates or tachysystole with changes in fetal heart rate^2^. Despite a higher incidence of tachysystole when induction was performed with misoprostol, this did not imply differences in cesarean section rates or neonatal outcomes^11^. In a meta-analysis published by Wang et al.^12^, including 8 studies with 1,669 pregnant women, the use of vaginal misoprostol showed less oxytocin augmentation when compared with dinoprostone. The other obstetric/neonatal outcomes, such as tachysystole, uterine hyperstimulation, vaginal delivery within 24 h, cesarean section, neonatal intensive care unit admission, and Apgar score at 5th min<7, were similar between the groups.

In our study, we included pregnant women with Robson Classification Groups 2 and 4 for labor induction with vaginal misoprostol. Most of the pregnant women included in our study belonged to Robson Group 2a (nulliparous), which is to be expected considering that the most common indication for induction of labor was a gestational age of>41 weeks. There was also a significant relationship between the Robson group and type of delivery, with multiparous women more likely to have a vaginal delivery. Vargas et al.^13^ performed a retrospective cohort study to assess the impact of induction of labor on cesarean section rates using the Robson Classification. We included 1,166 pregnant women, and the cesarean section rate was 20.9%. The highest cesarean section rate was observed in Robson Classification Groups 5 (65.2%) and 8 (32.3%). Robson Classification Group 2 was the highest contributor to the overall cesarean section rate, since it represented 56.7% of the pregnant women.

In our study, we evaluated 196 cases of pregnant women who underwent labor induction, of which 140 (71.4%) were successful and 56 (28.6%) were unsuccessful. Among the patients who successfully induced labor, 44 also used oxytocin in the induction/conduction of labor, while in the group of unsuccessful patients, 20 used oxytocin. In a retrospective cohort study, Berkley et al.^14^ evaluated the efficacy of labor induction with vaginal misoprostol (25 μg every 3–6 h) in nulliparous pregnant women with severe preeclampsia (145) and an unfavorable Bishop score. The rate of successful vaginal delivery was 65.5% (95). Vaginal delivery was associated with a shorter postpartum stay and less neonatal respiratory distress. In our study, using a majority low-risk population (75% without any risk factor), we obtained a higher rate of vaginal delivery (71.4%, 140/196). Yosef and Getachew^15^ performed a retrospective cross-sectional study with 294 mothers (undefined risk) who delivered in their service. The prevalence of labor induction was 20.4% (75% with oxytocin and 25% with vaginal misoprostol), and the most prevalent cause of induction was preeclampsia (41.6%). Of the 60 induced mothers, 23.3% had failed induction. In our study using a majority low-risk population, the main indication was gestational age>41 weeks (46.4%), and the preeclampsia indication occurred in only 6.6%; however, the rates of unsuccessful labor induction were higher in our study (28.6 vs. 23.3%).

In our study, no previous deliveries and the presence of one previous delivery were significant predictors of successful induction of labor with vaginal misoprostol. No previous delivery decreased the odds of successful induction of labor by 0.24 times, while one previous delivery increased the odds of successful induction of labor by 3.40 times. Caliskan et al.^16^ assessed the possible predictors of unsuccessful labor induction with vaginal misoprostol (50 μg each 6 h) in 1,030 pregnant women with single fetuses,>34 weeks of gestation, and Bishop score<5. Increasing gestational age in the Bishop score decreased the risk of unsuccessful labor induction. Corrêa et al.^17^ determined the predictive factors for the success of labor induction with vaginal misoprostol (1 tablet of 25 μg vaginally every 4 h for the first 5 doses and 2 tablets of 25 μg vaginally 50 μg every 6 h for the 6th, 7th, and 8th doses) in 873 high-risk pregnant women. The successful labor induction rate was 72% with vaginal delivery. They observed that maternal age<24 years, previous vaginal deliveries, lower gestational age, and greater cervical dilation were predictors of successful labor induction. We believe that none or one previous delivery were predictors of successful induction of labor because the majority of our sample consisted of nulliparous pregnant women.

In our study, a weak but significant negative correlation was observed between the Bishop score and the number of vaginal misoprostol tablets used. Drakopoulos et al.^18^ evaluated the number of oral misoprostol tablets needed to achieve a Bishop score of ≥6 in a retrospective study of 400 pregnant women. The incremental probability of achieving a significant change in Bishop score after 7 tablets was low (+2.0%). This study is consistent with our findings of a weak correlation between the Bishop score and the number of vaginal misoprostol tablets.

The strengths of this study were its prospective design, the fact that it was carried out in a referral maternity hospital for low-risk pregnancies, and the fact that it followed international recommendations for inducing labor with vaginal misoprostol. Possible limitations would be the relatively small sample size.

## CONCLUSION

We observed a high rate of labor induction success using vaginal misoprostol in a low-risk population, mainly in multiparous and with gestational age>41 weeks. No previous delivery decreased the success of labor induction, while one previous delivery increased the success of labor induction with vaginal misoprostol.
